# Misdiagnosis of malaria using wrong buffer substitutes for rapid diagnostic tests in poor resource setting in Enugu, southeast Nigeria

**DOI:** 10.5281/zenodo.10878928

**Published:** 2014-05-12

**Authors:** Johnbull S. Ogboi, Polycarp U. Agu, Adeniyi F. Fagbamigbe, Onyemocho Audu, Augustine Akubue, Ifeyinwa Obianwu

**Affiliations:** 1 Malaria & Human Development, Department of Life Sciences and Public Health, University of Camerino, 62032 Camerino (MC), Italy/RRI, Enugu, Nigeria.; 2 Department of Obstetrics and Gynaecology, University of Nigeria Teaching Hospital, Ituku-Ozalla, Enugu, Nigeria; 3 Department of Epidemiology and Medical Statistics, Faculty of Public Health, College of Medicine, University of Ibadan, Ibadan, Nigeria; 4 Department of Epidemiology and Community Health, College of Health Sciences, Benue State University, Makurdi, Nigeria; 5 Roll Back Malaria, World Health Organization, Zonal Office Enugu, Nigeria; 6 Department of Haematology and Immunology, College of Medicine, University of Nigeria, Enugu Campus, Nigeria

## Abstract

**Background:**

A key to the effective management of malaria is prompt and accurate diagnosis, and the use of malaria rapid diagnostic tests (mRDTs) is becoming relevant in the absence of reliable microscopy. This study explored the phenomenon of using the wrong buffer vial (often a kit from another brand or buffer from HIV rapid test kits), dextrose, saline or distilled water among health care providers who used RDTs for malaria diagnosis in resource poor settings in Enugu South East, Nigeria.

**Materials and Methods:**

Laboratory personnel (medical laboratory scientists, technicians, assistants, nurses, community health extension workers (CHEW), community health officers (CHO) and doctors) were interviewed using structured questionnaires and results were checked using the SOP checklist. The selection criterion was a prior experience with using RDTs, and any facility that did not use RDTs was excluded.

**Results:**

Of the 80 study participants that completed their questionnaires, 56.3% reported that malaria diagnosis was positive using non-buffer RDTs detection while others reported negative results. Among the various professionals who used RDTs, 76.2% reported to have run out of RDT buffer stock at least once. Of the study participants that ran out of RDT buffer solution, 73% declared to have used non-RDT alternatives (physiological saline, 0.9% NaCl), distilled water, HIV buffer or ordinary water). Only 30% had received formal training on the proper usage and application of RDTs while 70% had never received any formal training on RDTs but learnt the technique of using RDT on the job.

**Conclusions:**

This study demonstrated that at least three quarters of health care workers in a resource poor setting had run out of buffer when using malaria RDTs and that the majority of them had used buffer substitutes, which are known to generate inaccurate tests results. This has the consequence of misdiagnosis, thus potentially damaging the credibility of malaria control.

## 1 Introduction

Malaria case management remains a vital component of malaria control strategies [1]. Morbidity, mortality and transmission of malaria can be reduced if prompt and accurate diagnosis is available that will ensure correct treatment. Laboratory diagnosis of malaria has traditionally relied upon identification of malaria parasites in Giemsastained smears of peripheral blood. Recently, rapid diagnostic tests (RDTs) for the detection of *Plasmodium falciparum* infection were introduced in Nigeria to overcome problems associated with time constraints and the poor sensitivity in diagnosing malaria infections with a low level of parasitaemia by microscopy. They complement microscopy-based diagnosis where such services are not available. RDTs offer the potential of providing accurate and timely diagnosis of *P. falciparum* parasites, which are responsible for 98% of malaria infections in Nigeria.

RDTs are devices based on the detection of specific antigens (proteins) released from the parasitized erythrocytes. The simplest form is a dipstick (test strips placed in wells containing blood or buffer, in which the nitrocellulose strip may be placed in a plastic cassette or on a card that provide a useful guide to the presence of clinically significant malaria infection. The three main groups of antigens are histidine-rich protein 2 (HRP-2) specific to *P. falciparum,* parasite specific *Plasmodium* lactate dehydrogenase (pLDH) and aldolase (pan-specific). These RDTs display a control line and two or three test lines: one targeting *P. falciparum*-specific antigen, another line targeting antigens common to the four species such as pan specific *Plasmodium* and in case of so-called four band RDTs, a third line which targets *P. vivax*-specific pLDH (Pv-pLDH) [2].

In Nigeria, RDTs are currently rolled out by the National Malaria Control Programme (NMCP) in all settings as a tool for parasite-based diagnosis in the scope of artemisinin-based combination therapy (ACT). In the last four years, RDTs have improved technically for malaria diagnosis, especially in rural communities when compared with the gold standard (microscopy). However, despite their ease and simplicity, they are not completely fail proof [3,4]. RDTs that are marketed in Nigeria include materials for 20-25 tests with lancets for finger pricking, test strips (cassette, dipstick), transfer devices (pipettes, inverted cup loop, capillaries or loop) and buffer. All materials are equivalent to the number of tests per device. Every RDT requires a buffer, supplied either in a single bottle or dropper vial, to help lyse the blood, and to allow capillary flow (lateral diffusion or immunochromatographic separation) along the nitrocellulose strip. The immunochromatographic technology remains the common basis for all practical malaria RDTs under consideration at this time. RDTs require minimal training for usage among health workers in any setting.

When performing the RDTs with the dedicated buffer provided in the kit, most problems arise when the buffer bottle (vial) is lost, for instance when used for bedside testing in the emergency room and not put back in the RDT box. The use of more drops of buffer (volume) by health care practitioners as prescribed by the manufacturer is also an error that causes inaccurate results. To compensate for this, health care providers therefore use either a buffer vial from another kit (often a kit from another brand or buffer from HIV rapid test kits), dextrose, saline or distilled water. Based on this wrong application and practice, this study therefore explored the phenomenon among health care providers (laboratory personnel – medical laboratory scientists, technicians, assistants, nurses, community health extension workers (CHEW), community health officers (CHO) and doctors) on the usage of RDT buffer solutions or alternatives in resource poor settings of Enugu South East, Nigeria.

## 2 Materials and Methods

### 2.1 Study area

Enugu State is an inland State in South East Nigeria. It shares boundaries with Anambra to the West, Abia State to the South, Kogi to the North and Benue to the North East and Ebonyi to the East. In the 2006 Population and Housing Census, Enugu state population consisted of 1,596,042 males and 1,671,795 females [5]. Enugu State has rich agricultural land as a result of its location within the tropical forest and savannah belts. The humid climate and the almost year-round distribution of rainfall allow the breeding of mosquitoes throughout the year.

### 2.2 Study participants

A health facility-based cross-sectional descriptive study design was employed from May 2013 to September 2013 at Enugu State (urban and rural) communities. The study captured all Local Government Areas where RDTs are used for malaria diagnosis in an endemic region with high malaria transmission. The predominant malaria parasite species is *P. falciparum.* A total of 85 health facilities in Enugu State, including government-owned facilities, private clinics/hospitals, mission hospitals and stand-alone laboratories, were selected. The criterion of selection was a prior experience with using RDTs, and any facility that did not use RDTs was excluded.

The study involved 85 health care practitioners (one per facility was randomly selected), comprising laboratory personnel (medical laboratory scientists, technicians, assistants, nurses, CHEW, CHO and doctors) using RDTs. Participants were interviewed by trained research assistants using structured administered questionnaires on the usage of RDT buffer in malaria diagnosis and their practice, especially when the buffer solution is out of stock, or their use of alternatives with different RDT kits in their health facility. The RDTs results performed as part of routine patient care were also checked.

### 2.3 RDTs used in the study area

Three types of RDTs recommended by the World Health Organization (WHO) are commonly used in the study area and were assessed in this study: (1) cassette format of one-step malaria Antigen P.f., produced by Standard Diagnostic, Korea (Promedt Consulting, GmbH Germany); (2) Paracheck® cassette format for P.f. (Orchid Biomedical Systems, India); (3) The cassette format of Core malaria PfTM (Core Diagnostics, UK).

### 2.4 Data analysis

The data were encoded anonymously into Excel work-sheets and checked by an independent expert. The database was further cleaned and converted into Statistical Package for Social Sciences (SPSS) version 17 (Illinois, Chicago), which was used for the data analysis. The outcome variable was usage of RDT buffers while the independent variables included age, sex, education, location, experience, type of facility and training of the respondents. We also considered type of RDT and buffer solution used, whether it was out of stock and the result of the RDTs results performed as part of routine patient care. We carried out descriptive statistics and Chi-square tests of association with statistical significance set at 5%.

### 2.5 Ethical Considerations

Informed consent was sought from the respondents. The ethical committees concerned in the Enugu State were contacted and ethical clearance was granted for the conduct of the study.

## 3 Results

**Fig 1:**
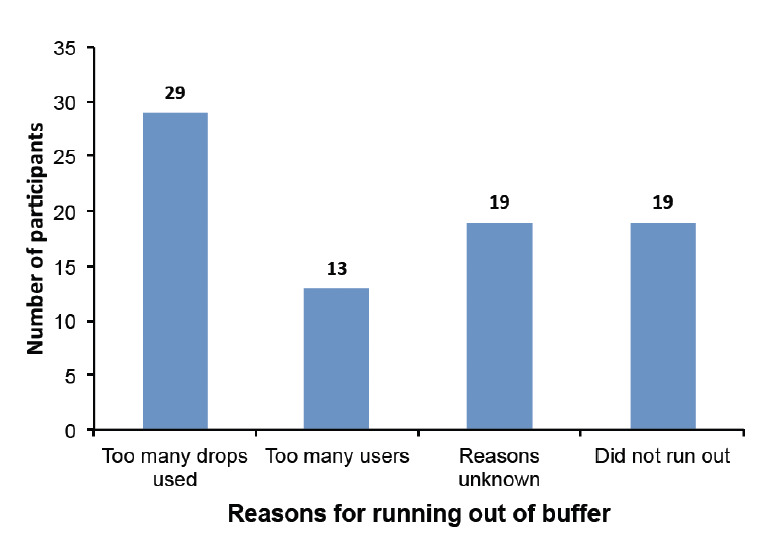
Reasons given why health care workers ran out of buffer.

Table 1 shows the breakdown of variables in different categories with percentages. Most of the health facilities (52 out of 85, 65%) were located in urban centres, while 28 (35%) were located in rural communities. At the various health facilities, 85 health care practitioners, including laboratory personnel (medical laboratory scientists, technicians, assistants, nurses, CHEW, CHO and doctors) were enrolled in the study. Five questionnaires were rejected due to errors and incompleteness. Of the 80 questionnaires analysed, 72.5% of the participants were males and 27.5% females, with a mean age of 35.5±6.4 years. In this study, 38.8% of participants had secondary education while 61.2% had tertiary education. Out of the 80 study participants who completed their questionnaires, the positivity rate prevalence of malaria as reported using RDTs detection was 56.3%, compared with the national positivity rate prevalence of 23.6% around the study zone (South East Nigeria) [6], while 43.7% reported a negative result. Among the various professionals who used RDTs, 76.2% reported to have run out of RDT buffer stock solution at least once, while 23.8% had never ran out of RDT buffer solution. Of those who had ran out of RDT buffer solution, 73% declared to have used non-RDT alternatives, such as physiological saline (0.9% NaCl), distilled water, HIV buffer and ordinary water from a borehole source. Among the facilities declaring to have used non-RDT buffer, 33.9% were private or standalone laboratories, 27.1% were private clinics/hospitals, 22% were missions, 11.9% were private health clinics (PHCs) and 5.1% were government hospitals (Table 2). Various reasons were given for why the RDT buffer did not last in their facilities: presumed too many drops/too much volume being used 29 (36.3%), reason unknown 19 (23.8%) and too many users 13 (16.3%), while the remaining 19 (23.8%) never ran out of RDT buffer (Figure 1). On the aspect of training how to use RDT kits in the detection of malaria, only 30% had received formal training on the proper usage and application of RDTs. The majority (73.8%) learned how to use RDT on the job. Table 3 shows the relationship between sociodemographic variables (age, sex, education level, profession, location and years of experience) and the use of RDT buffers, but no significant differences in the usage of non-RDTs were found. Table 4 compares the relationships be-tween malaria-related variables (years of using RDTs, facility type, test results, RDT brand and RDT usage training) and the use of RDT buffers. Government-owned hospitals and PHCs used RDT buffers more frequently than other health facilities. The usage of RDT buffers was significantly associated (Chi-square test, *P* value = 0.022) with the types of health facilities visited.

**Table 1.** Distribution of socio-demographic and malaria-related variables among the respondents (*n*=80).

Variable and categories	Number of participants (%)
**Age**	
25-34	34 (42.5)
35 and above	46 (57.5)
**Sex**	
Male	58 (72.5)
Female	22 (27.5)
**Education**	
Secondary	31 (38.8)
Tertiary	49 (61.2)
**Years of using RDT**	
1	43 (55.0)
2 or 3**	36 (45.0)
**Profession**	
CHEW/CHO	11 (13.8)
Doctor/ Nurse ***	9 (11.3)
Med. Laboratory Scientist	21 (26.3)
Laboratory Assistant	13 (16.3)
Laboratory Technician	26 (32.5)
**Types of facility**	
Clinic/Hospital	21 (26.3)
Private lab or Standalone	27 (33.8)
Mission Hospital	16 (20.0)
PHC	10 (12.5)
State Government Hospital	5 (6.3)
Tertiary Hospital*	1 (1.3)
**Location**	
Rural	28 (35.0)
Urban	52 (65.0)
**Brand of RDT in use**	
Core	8 (10.0)
Paracheck	29 (36.3)
SD Bioline	43 (53.8)
**Buffer out-of-stock at least once**	
Yes	61 (76.2)
No	19 (23.8)
**Reason for running out of buffer**	
Presumed too many drops	29 (36.3)
Too many hands using	13 (16.3)
Don’t know	19 (28.3)
Did not run out of buffer	19 (23.8)
**RDT Result**	
Positive	45 (56.3)
Negative	35 (43.7)
**Received any training on RDT**	
No	56 (70.0)
Yes	24 (30.0)
**Learnt how to do RDT on the**	
**job**	21 (26.3)
No	59 (73.8)
Yes	
**Buffer used**	
RDT Buffer	19 (23.8)
Non-RDT buffer	59 (73.8)
No Action taken	2 (2.4)

* One tertiary hospital included;

** Three respondents had been using RDTs for three years;

*** Three doctors included.

**Table 2.** Usage of RDT and non-RDT buffer in different health facilities.

Facility	RDT usage (%)	Non-RDT usage (%)
Private Clinic	11 (61.1)	16 (27.1)
Government Hospital	2 (11.1)	3 (5.1)
Mission hospital	3 (16.7)	13 (22.0)
PHC	2 (11.1)	7 (11.9)
Private/Standalone labs	0	20 (33.9)
Total	18 (100)	59 (100)

**Table 3.** Relationships between socio-demographic variables and use of RDT buffers.

Variable and categories	RDT usage (%)	Non-RDT usage or alternatives (%)	*P* value (Chi-square test)
**Age**			0.341
25-34	20.6	79.4	
35 and above	27.3	72.7	
**Sex**			0.458
Male	27.3	76.8	
Female	23.2	72.7	
**Education**			0.546
Secondary	23.3	76.7	
Tertiary	25.0	75.0	
**Profession**			0.497
CHEW/CHO	27.3	72.7	
Doctor/Nurse*	37.5	62.5	
Med. Laboratory Scientist	30.8	69.2	
Laboratory Assistant	8.3	91.7	
Laboratory Technician	19.0	81.0	
**Location**			0.177
Rural	32.1	67.9	
Urban	20.0	80.0	
**Years of experience**			0.147
< 5 years	27.3	72.7	
5 - 9 years	33.3	66.7	
> 9 years	12.9	67.1	

* Included only two doctors.

## 4 Discussion and Conclusions

The changing epidemiology of malaria due to scale up interventions and the introduction of ACTs have increased the urgency of improving the specificity of malaria diagnosis. In recent years, new technological methods have been evaluated as alternatives to microscopy, and given the absence or poor execution of microscopy, alternative diagnostic strategies are needed, especially in areas where malaria is highly endemic. One of these strategies includes malaria antigen detection using RDTs. The increasing burden of malaria disease, the emergence of resistance to antimalarial agents and the recent deployment of expensive ACTs into regions where malaria is highly endemic have also increased the need for rapid and accurate diagnosis of patients who may be infected with malaria. RDTs have been reported to be useful and easy tools for malaria diagnosis in towns, hard to reach communities and villages especially in our poor resource settings. However, the use of substitutes for the RDT buffer may give rise to false positive and negative results [7], as the present study showed that replacing malaria RDT buffers with (normal saline (0.9% NaCl), distilled water, HIV buffer and ordinary water) may have cause inaccurate result with 56.3% reporting that malaria diagnosis was positive using non buffer RDTs detection kits (Table 1). False positive and false negative results might delay/exclude true diagnosis, with consequences that could lead to the death of patients. The consequence of false positive RDTs using alternative solutions is the unjustified prescription of ACT treatment, which might lead to resistance. This may account for our failure to control malaria in many communities and the consequent increasing mortality.

In RDTs, blood and buffer are added to the strip where the lysing agent and labelled antibodies are located and are drawn into the strip. If antigen is present, labelled antibody -antigen complexes will be trapped on the test line and become visible. Additional indicator-labelled antibodies are positioned on the control line and becomes visible. The substitution of the buffer may contribute to non-specific binding of the conjugate to the capture antibody either by reducing pH and ionic strength, thereby allowing non-specific bindings, or by slowing the lateral diffusion or immunochromatographic separation along the nitrocellulose strip, which in turn reduces flowing of non-specific interactions. This study is consistent with previous findings [8], which stated mechanical issues: during application of the capture antibody, the dispensing pipette may carve an indentation into the membrane, with an additional decrease in capillary flow rate. Furthermore, as the buffer helps to ensure optimal conditions of pH and ionic strength, there is also a concern of possible false-negative results.

**Table 4.** Relationship between malaria-related variables and use of RDT buffers.

Variable and categories	RDT usage (%)	Non-RDT usage or alternatives (%)	*P* value (Chi-square test)
**Years of using RDT**			0.142
1	27.3	72.7	
2	33.3	66.7	
3	12.9	87.1	
**Facility type**			0.022
Clinic	30.0	70.0	
Government hospital	40.7	59.3	
Mission	18.8	81.3	
PHC	40.0	60.0	
Private laboratory	0.0	100.0	
**Brand of RDT**			0.999
Core	25.0	75.0	
Paracheck	24.1	75.9	
SD Bioline	24.4	75.6	
**Result**			0.291
Negative	29.5	70.5	
Positive	17.6	82.4	
**Received any RDT training**			0.999
No	23.6	76.4	
Yes	26.1	73.9	
**Learnt RDT on the job**			0.539
No	31.6	68.4	
Yes	22.0	78.0	

The performance of malaria RDTs can also be adversely affected during transportation in the rural tropics, especially in our environment with high temperatures above 38°C. Given that temperature, time, humidity or windy conditions can rapidly degrade nitrocellulose capillary flow action of malaria RDTs, it is possible that this could lead to an unfolding of binding sites of antibodies and leading to false results as suggested by other studies [6, 8]. Although proper packaging can mitigate the degrading effects of these factors during storage, once the test kit has been removed from its packaging, it becomes rapidly vulnerable to them. Recognising these facts requires adequate training, which is supported by findings from previous studies [8-10].

However, our study revealed that the practice of applying too many drops of buffer was the main reason why many health practitioners ran out of buffer (36.3%), while the rate of too many users using it at the same time was 16.3% (Figure 1). A lack of training in 70% of participants was also associated with improper usage of RDTs despite the fact that RDTs require minimal training. This lack of training can have potentially important negative implications for malaria control programmes in Nigeria, especially for the application of buffer required for a single RDT test.

Every organisation is determined to survive and prosper in the current challenging economy in the control and elimination of malaria, and must therefore understand the imperative to invest in reviewing the existing training programs and to assess whether the issue of (i) use of the correct number of buffers and (ii) buffer replacement should be considered with professional development in order to improve efficiencies in service delivery. This has become especially apparent now that we have discovered that too many drops were being used during routine analyses. The use of RDTs is very helpful for the effective use of antimalarial drugs as treatment is based on parasite diagnosis and not only fever. Parasitological confirmation of the malaria through microscopy is part of good clinical practice that should always be part of malaria case management [11,12]. Therefore, the prevention of buffer substitution could be addressed by proper training on the usage and by providing more than one buffer vial per RDT kit at all levels of health care organization, as shortage and replacement of buffer vials is a common problem in resource limited settings like ours. This is highlighted by the finding that government-owned hospitals tended to use RDT buffers more frequently than other health facilities (Table 4). The issue of buffer substitution should further be addressed in RDT instructions to create awareness, since many health or village workers without formal medical laboratory training or with minimal training are using it [8]. It is recommended that a trained laboratory officer should supervise use of the correct buffer.

## Supplementary Information

Misdiagnosis of malaria using wrong buffer substitutes for rapid diagnostic tests in poor resource setting in Enugu, South East, Nigeria

### Questionnaire

1. Name of Facility ……………………………...
2. Type of facility ……………………………….
3. Age ……………………………………………
4. Sex: Male ( ) Female ( )
5. Profession: Medical laboratory scientists ( ) Medical laboratory technicians ( ) Medical laboratory assistants ( ) Nurses ( ) community health extension workers ( ) community health officers ( ) Medical doctors ( )
6. Educational level: Secondary ( ) Tertiary ( )
7. How long have you been working in this facility? less than 5 yrs ( ) 5-9 years ( ) above 9 years ( )
8. Facility location: Urban ( ) Rural ( )
9. Do you use RDTs? Yes ( ) No ( )
10. Brand of RDTs use: ……………………………
11. How long have you been using RDTs? 1 year ( ) 2 years ( ) 3 years and above ( )
12. Have you at any point run out of RDT buffer? Yes ( ) No ( )
13. What kind of buffer do you used RDT buffer ( ) non RDT buffer ( ) no action taken ( )
14. If non-RDT buffer, what did you use? Kit from another brand or buffer from HIV rapid test kits ( ) dextrose ( ) physiological saline or distilled water ( )
15. What is your reason for running out of buffer stock? Too much volume ( ) loss of RDT buffer vial ( ) usage by many hands ( ) don’t know ( )
16. What was the result of the malaria RDT? Positive ( ) Negative ( )
17. Have you received nay RDTs training? Yes ( ) No ( )
18. Where did you learn how to run RDTs? On the job ( ) School ( ) Training ( )
